# Bioactive natural alkaloid 6−Methoxydihydrosanguinarine exerts anti−tumor effects in hepatocellular carcinoma cells via ferroptosis

**DOI:** 10.3389/fphar.2025.1500461

**Published:** 2025-04-24

**Authors:** Linfen Han, Chengchang Gao, Xiaorui Jin, Yingping Li, Liangjie Chen, Donglin Li, Qinqin Deng, Xueli Bian

**Affiliations:** ^1^ The MOE Basic Research and Innovation Center for the Targeted Therapeutics of Solid Tumors, Department of Nutrition, The Second Affiliated Hospital, Jiangxi Medical College, Nanchang University, Nanchang, China; ^2^ School of Basic Medical Sciences, Jiangxi Medical College, Nanchang University, Nanchang, China; ^3^ Shanxi Academy of Advanced Research and Innovation, Taiyuan, China

**Keywords:** 6-methoxydihydrosanguinarine, HCC, ROS, ferroptosis, GPX4

## Abstract

**Introduction:**

Ferroptosis is a form of regulated cell death driven by the accumulation of iron–dependent lipid peroxides, and ferroptosis–mediated cancer therapy has gained considerable attention. Despite emerging evidence that ferroptosis induction effectively suppresses hepatocellular carcinoma (HCC) progression and enhances chemosensitivity, the development of resistance to ferroptosis‐targeting therapies remains a critical challenge. Natural active compounds have great potential in cancer treatment.

**Methods:**

The impact of 6-ME on the cell viability of HCC cells was assessed using the Cell Counting Kit-8 (CCK‐8) assay and colony formation assay. Furthermore, cellular morphology of HCC cells was visualized under inverted fluorescence microscopy. Intracellular reactive oxygen species (ROS) and lipid peroxidation levels were quantified using fluorescence probes and determined by flow cytometry analysis. The expression of ferroptosis-related proteins and genes was determined via Western blot and quantitative real-time PCR analyses.

**Results:**

Here, we demonstrate that 6–Methoxydihydrosanguinarine (6–ME), an alkaloid from *Macleaya cordata*, exerts anti–tumor functions in HCC cells via ferroptosis. Stimulation with 6–ME induces intracellular ROS production, cell growth inhibition, and cell death in HCC cells, and these effects can be weakened by the ROS scavenger GSH or NAC and ferroptosis inhibitors deferoxamine mesylate (DFO) or ferrostatin–1 (Fer–1). Mechanistically, 6–ME downregulates the expression of the key ferroptosis defense enzyme GPX4 at the transcriptional level, leading to excessive lipid peroxidation and ferroptosis in HCC cells. Importantly, low concentrations of 6–ME also enhanced the ferroptosis sensitivity induced by RSL3 and IKE in HCC cells.

**Conclusion:**

These findings reveal that the natural product 6–ME exerts anti–tumor functions in HCC cells *via* ferroptosis and underscore the potential of 6–ME administered alone or in combination with canonical ferroptosis inducers for the treatment of HCC patients.

## 1 Introduction

Liver cancer is a highly invasive malignancy with a dismal prognosis. According to the National Cancer Center in February 2024, the annual incidence and mortality rates of liver cancer in China rank fourth and second respectively, with over 316,500 deaths each year (Han et al., 2024). Hepatocellular carcinoma (HCC) constitutes approximately 75%–85% of primary liver cancer cases (Zhou et al., 2019). Despite substantial advancements in the development of novel chemotherapeutic agents and therapeutic strategies, the management of HCC continues to pose considerable challenges due to severe adverse effects and the emergence of drug resistance (Zhang et al., 2020; Yang et al., 2024). Therefore, there is an urgent need to develop safe and effective therapeutic agents for the treatment of HCC.

Ferroptosis is a newly identified form of programmed cell death that differs from apoptosis, necrosis, and autophagy (Dixon et al., 2012; Li et al., 2024). The primary mechanism underlying ferroptosis involves the catalyzed accumulation of reactive lipid peroxidation products due to the action of divalent iron and lipoxygenases, leading to the high expression of polyunsaturated fatty acids (PUFAs) within the cell membrane (Stockwell, 2022). Uncontrolled lipid peroxidation serves as a prominent indicator of ferroptosis. Ferroptosis is characterized by a reduction in glutathione peroxidase 4 (GPX4), a critical regulatory enzyme of the antioxidant defense system, specifically the glutathione (GSH) system (Wu et al., 2023; Yu et al., 2024). In the classical regulatory pathway of ferroptosis, cystine is transported into the cell *via* the cystine/glutamate antiporter (system Xc−) located in the plasma membrane, facilitating the synthesis of GSH, an essential intracellular reducing agent (Sato et al., 1999). GPX4 functions as the primary enzyme responsible for neutralizing Phospholipid Hydroperoxides (PLOOHs). GPX4 catalyzes the reduction of PLOOHs to their corresponding alcohols (PLOHs) in a selenium (Se)−dependent reaction, providing protection against ferroptosis (Maiorino et al., 2018). It has been reported that certain small−molecule compounds, such as RSL3, can directly inhibit GPX4, while others, like IKE, impair cystine uptake (Tschuck et al., 2023). Consequently, inhibition or depletion of GPX4 leads to the accumulation of PLOOHs, resulting in irreparable damage to cellular membrane structures and inducing ferroptosis (Jiang et al., 2021). Emerging evidence suggests that ferroptosis is closely associated with various diseases, including cancer, neurodegenerative disorders, infections and inflammatory conditions (Stockwell et al., 2017). According to the Global Cancer Statistics 2022 published in 2024, with nearly 870,000 new cases and over 750,000 deaths worldwide in 2022, liver cancer being one of the leading causes of cancer incidence and mortality (Bray et al., 2024). Primary liver cancer primarily consists of HCC. Despite the availability of numerous therapeutic interventions for HCC, clinical outcomes remain suboptimal. Given the unique role of ferroptosis as a regulated mode of cell death, it represents a promising target in HCC therapy (Nie et al., 2018). Thus, a comprehensive understanding of the regulatory networks governing ferroptosis in liver cancer is essential for the development of novel treatment strategies.

*Macleaya cordata*, a perennial herb of belonging to the Papaveraceae family, exhibits significant therapeutic effects against tumors and in the improvement of liver function (Liu et al., 2022). 6−Methoxydihydrosanguinarine (6−ME) is an isoquinoline alkaloid derived from the extracts of *M. cordata.* 6−ME possesses antimicrobial properties, displaying a potent inhibitory effect against *Staphylococcus aureus* (Xue et al., 2017). Previous investigations have reported that 6−ME can inhibit the PI3K/AKT/mTOR signaling pathway through ROS accumulation, thereby inducing apoptosis and autophagy in MCF−7 cells. Additionally, 6−ME has been shown to influence the growth dynamics of HT29 and HepG2 cells (Yin et al., 2005). The cytotoxicity of 6−ME is manifested by upregulation of DR5 induced by ROS, making cells sensitive to TRAIL−mediated apoptosis, thereby causing HCC cell cycle arrest, DNA damage, and apoptosis (Wang et al., 2023). In conclusion, current experimental evidences on 6−ME’s modulation of the PI3K/AKT/mTOR signaling pathway, DR5 upregulation, and induction of cell cycle arrest/DNA damage collectively delineate mechanistic pathways by which 6−ME induces ROS accumulation, which could contribute to lipid peroxidation, a hallmark of ferroptosis. However, whether 6−ME combats HCC by promoting ferroptosis has not been fully elucidated.

In this study, we revealed the cytotoxic effects of 6−ME on HCC cells and elucidated its underlying mechanisms. Our findings suggest that 6−ME significantly impedes the proliferation of HCC cells and induces ferroptosis through the downregulation of GPX4.

## 2 Materials and methods

### 2.1 Cell lines and cell culture

Human hepatoma cell lines (HLE and HCCLM3) and normal hepatic stellate cells (LX−2) were purchased from the cell bank of the Chinese Academy of Sciences. All cells were cultured in Dulbecco’s Modified Eagle’s Medium (DMEM, Solarbio, Cat# 11965) supplemented with 10% fetal bovine serum (FBS, Excell Bio, FSP500). All the cell lines were maintained at 37°C in a humidified atmosphere containing 5% CO_2_.

### 2.2 Reagents

Reagents: 6−Methoxydihydrosanguinarine (TargetMol, Cat# T8724); Cell lysis buffer (CST, Cat# 9803S); Protease inhibitor PMSF (Wuhan Dingguo Biotechnology, Cat# 329−98−6); Propidium iodide (PI, TargetMol, Cat# T2130); Paraformaldehyde (Solarbio, Cat# P1110); N−acetylcysteine (NAC, MCE, Cat# HY−B0215); Reduced L−glutathione (GSH, MCE, Cat# HY−D0187); Deferoxamine mesylate (DFO, TargetMol, Cat# T1637); Ferrostatin−1 (Fer−1, TargetMol, Cat# T6500); DCFH−DA (MCE, Cat# HY−D0940); C11−BODIPY 581/591 (MCE, Cat# HY−D1691); RSL3 (CSNpharm, Cat# CSN17581); imidazole ketone erastin (IKE, TargetMol, Cat# T5523); TransZol Up reagent (TransGen, Cat# ET111−01−V2); M5 Super plus qPCR RT kit with gDNA remover (Mei5bio, Cat# MF166−plus−T); M5 HiPer one−step RT−PCR kit (Mei5bio, Cat# MF051−01); ABI QuantStudio 7 Flex with SYBR kit (TransGen, Cat# AQ601−02); Cell Counting Kit−8 (CCK−8, TargetMol, Cat# C0005), CHX (MCE, Cat# HY−12320); MG132 (Biovision, Cat# 1791−5); Transfection reagent (Pufei Biotech, Cat# 2102−100).

### 2.3 Cell viability assay

HLE or HCCLM3 cells were resuspended to a density of 1 × 10^4^ cells/well and then seeded in 96−well plates. Following cell adherence, 6−ME were added, or along with the following treatments: 5 mM NAC, 5 mM GSH, 2 µM Fer−1, or 50 µM DFO. After 12 h, 10 µL CCK−8 solution was added to each well. After 1 h incubation, the optical density (OD) was measured at a wavelength of 450 nm using a microplate reader.

### 2.4 Colony formation assay

HLE or HCCLM3 cells were seeded in 6−well plates and allowed to adhere for 24 h under standard culture conditions. After confirming cell attachment, the cultures were treated with varying concentrations of 6−ME alone or in combination with 2.5 μM RSL3 or 10 μM IKE. Post−treatment, cells were fixed with 4% paraformaldehyde (PFA) at room temperature for 15 min, followed by three washes with PBS to remove residual fixative. Colonies were subsequently stained with 0.1% crystal violet solution for 5 min, rinsed thoroughly with distilled water to eliminate unbound dye, and air−dried. Finally, colony formation was photographed.

### 2.5 Assessment of cell morphology

HLE and HCCLM3 cell lines were seeded into 6−well plates and incubated for 24 h in a humidified atmosphere (37°C, 5% CO_2_) to ensure adherence. Cells were then treated with indicated concentrations of 6−ME, either alone or in combination with the following agents: 5 mM NAC, 5 mM GSH, 2 μM Fer−1, 50 μM DFO, 2.5 μM RSL3, or 10 μM IKE. Following the incubation period, cellular morphological alterations were assessed using an inverted fluorescence microscope and images were captured.

### 2.6 Cell death assay

HLE or HCCLM3 cells were seeded into 6−well plates. After cell adherence, cells were treated with various reagents for the indicated time. Following treatment, both adherent and floating cells were collected and stained with 10 μg/mL propidium iodide (PI). The percentage of dead cells was then analyzed using flow cytometry and FlowJo 10 software.

### 2.7 ROS and lipid ROS determination

The cells in the logarithmic growth phase were inoculated into 12−well plates at 50% confluence. Cells were treated with or without 6−ME for 4 h. Then 10 μM DCFH−DA (for total ROS level detection) or 10 μM C11−BODIPY581/591 probe solution (for lipid ROS level detection) was added to each well and incubated for 1 h, and then cells were harvested and analyzed by flow cytometry.

### 2.8 Western blot

Cells were lysed in lysis buffer (1% Triton, 20 mM Tris, 150 mM NaCl, 1 mM EDTA, 1 mM EGTA and 2.5 mM Sodium pyrophosphate) containing 1% PMSF. The samples were then subjected to SDS−PAGE for protein separation, and the target proteins were transferred to a PVDF membrane. The membrane was subsequently blocked with 5% non−fat milk. After blocking, the membrane was incubated with the corresponding primary antibody and the appropriate secondary antibody. The following antibodies were used: Vinculin (Santa Cruz, Cat# sc−73614, 1:10,000); GPX4 (Proteintech, Cat# 67763−1−Ig, 1:2000); SLC7A11 (Proteintech, Cat# 18790−1−AP, 1:3,000); FSP1 (Santa Cruz, Cat# sc−377120, 1:3,000); ACSL4 (Proteintech, Cat# 22401−1−AP, 1:8,000); HRP conjugated goat anti−mouse or rabbit antibodies (Santa Cruz, 1:6,000). Finally, the ECL chemiluminescent reagent was utilized for signal development.

### 2.9 RNA extraction and RT−qPCR analysis

Total RNA was extracted from cells using TRIzol reagent. The RNA was then reverse transcribed into cDNA using the M5 HiPer One−step RT−PCR Kit. Real−time quantitative PCR (RT−qPCR) analysis was performed using the ABI QuantStudio 7 Flex and the SYBR Green assay kit. β−Actin was used as the reference gene for normalization in these experiments. The following primers were used for RT−qPCR: GPX4, 5'−GAGGCAAGACCGAAGTAAACTAC−3′ and 5'−CCG​AAC​TGG​TTA​CAC​GGG​AA−3'; β−Actin, 5'−GTCACCAACTGGGACGACA−3′ and 5'−CAC​AGC​CTG​GAT​AGC​AAC​G−3'.

### 2.10 Transient transfection of plasmid

When HLE cells reached 70−80% confluence, transient transfection was performed using a lipid-based transfection reagent. Briefly, two sterile microcentrifuge tubes were prepared as follows: (A) 100 μL of serum−free DMEM containing 2 μg of plasmid DNA (pcDNA3.1−Flag or pcDNA3.1−GPX4−Flag) and (B) 100 μL of serum−free DMEM mixed with 6 μL of transfection reagent. The contents of tube B were gently combined with tube A, followed by vortexing for 10 s and incubation at room temperature for 15 min to allow transfection complex formation. The resulting mixture was then dropwise added to the cell culture and evenly distributed by gentle rocking. After 6 h of incubation at 37°C in a humidified 5% CO_2_ atmosphere, the transfection medium was replaced with complete growth medium (DMEM supplemented with 10% fetal bovine serum). At 36 h post−transfection, cells were exposed to varying concentrations of 6−ME for 12 h. Cell viability was subsequently quantified using the CCK−8.

### 2.11 Statistical analysis

All quantitative data represent the mean ± standard deviation (SD) of at least three independent experiments. Differences between two groups were evaluated using the unpaired Student’s *t*−test, while differences among multiple groups were analyzed by one−way ANOVA. The statistical significance across different conditions was evaluated by calculating *p* values (with a 95% confidence interval) using GraphPad Prism 9.0 software. *p ≤ 0.05; **p ≤ 0.01; ****p ≤ 0.0001; ns, not significant.

## 3 Results

### 3.1 6−ME inhibits HCC cell growth

**FIGURE 1:**
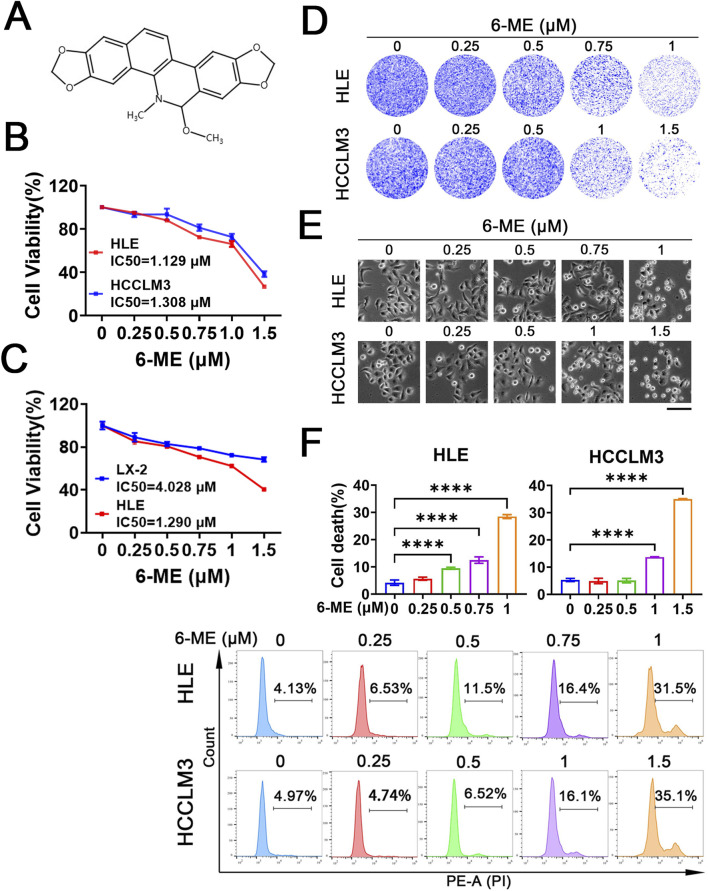
6−ME inhibits HCC cell growth. **(A)** Chemical structure of 6−ME. **(B, C)** HLE, HCCLM3 and LX-2 cells were treated with various concentrations of 6−ME (0, 0.25, 0.5, 0.75, 1, and 1.5 μM) for 12 h. Cell viability was assessed using the CCK−8 assay, and the IC50 values were calculated (n = 3). **(D)** Following a 12−hour treatment with a gradient of 6−ME concentrations (0, 0.25, 0.5, 0.75, and 1 μM for HLE cells; 0, 0.25, 0.5, 1, and 1.5 μM for HCCLM3 cells), cell proliferation was evaluated through crystal violet staining to assess colony−forming ability. **(E)** The morphological changes in HLE and HCCLM3 cells subjected to varying concentrations of 6−ME were examined, scale bar: 100 μm. **(F)** HLE and HCCLM3 cells were treated with different concentrations of 6−ME for 12 h, followed by PI staining and analysis via flow cytometry (n = 3). One−way ANOVA was used for statistical analysis. Data are represented as mean ± SD. Statistical significance was evaluated with ****p < 0.0001.

Previous studies have established the anti−proliferative efficacy of 6−ME (Figure 1A) in human lung adenocarcinoma A549 cells (Liu et al., 2024). To systematically evaluate its therapeutic potential in HCC, we investigated the dose−responsive effects of 6−ME on the viability of HLE and HCCLM3 cells. Cells were treated with escalating concentrations of 6−ME for 12 h, and viability was quantified *via* CCK−8 assay. The results demonstrated a significant, dose−dependent reduction in viability for both HCC cell lines, with calculated IC50 values of 1.129 μM (HLE) and 1.308 μM (HCCLM3) (Figure 1B). Importantly, the IC50 value of 6−ME in normal hepatic stellate cells (LX−2) was significantly higher than that in HLE cells (Figure 1C). Based on these findings, subsequent experiments used 1 μM 6−ME for HLE and 1.5 μM 6−ME for HCCLM3, corresponding to near IC50 concentrations. Further mechanistic analysis revealed that 6−ME treatment profoundly suppressed HCC cell proliferation, as evidenced by attenuated colony−forming capacity (Figure 1D) and morphological aberrations, including cellular shrinkage and increased apoptotic body formation (Figure 1E). Flow cytometric quantification confirmed a dose−dependent induction of cell death in both HLE and HCCLM3 cells, with higher 6−ME concentrations correlating with elevated cell death rates (Figure 1F). Collectively, these findings suggest that 6−ME effectively inhibits cell proliferation and induces cell death in HCC cells.

### 3.2 6−ME induces HCC cell death via ROS

**FIGURE 2:**
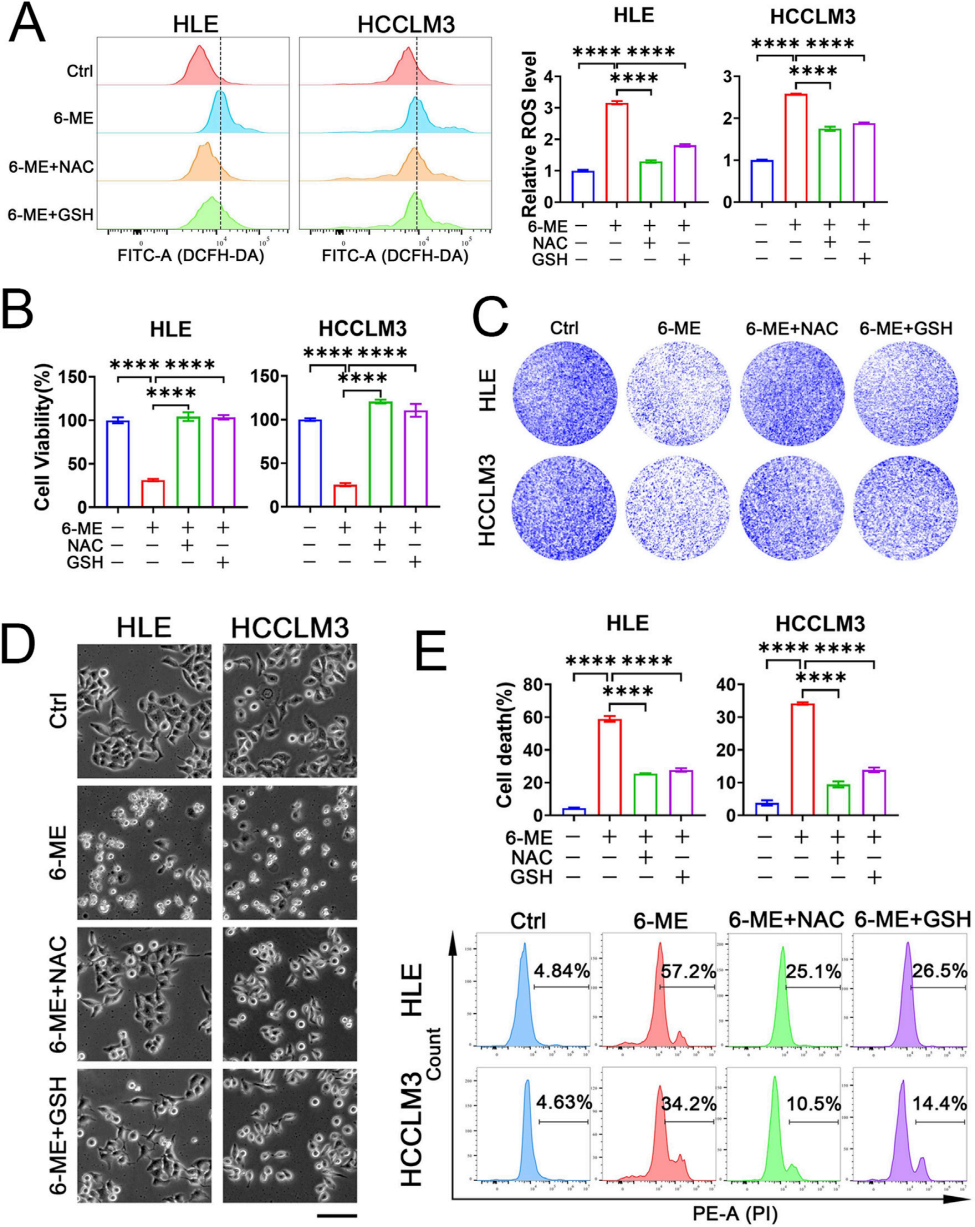
6−ME induces HCC cell death via ROS. **(A−E)** HLE and HCCLM3 cells were treated with or without 6−ME (1 μM for HLE and 1.5 μM for HCCLM3) in the presence or absence of 5 mM NAC or 5 mM GSH for 4 h **(A)** or 12 h **(B−E)**. **(A)** Cells were incubated with 10 μM DCFH−DA for 1 h to detect ROS *via* flow cytometry (n = 3). **(B)** Cell viability was assayed with CCK−8 (n = 3). **(C)** Clonogenic potential was evaluated through crystal violet staining. **(D)** Cellular morphology was observed under an inverted fluorescence microscopy, scale bar: 100 μm. **(E)** Following PI staining, cell death was further examined *via* flow cytometry (n = 3). One−way ANOVA **(A, B, E)** was used for statistical analysis. Data are represented as mean ± SD. Statistical significance was denoted as ****p < 0.0001.

Elevated levels of ROS have been implicated in the induction of cellular apoptosis (Glorieux et al., 2024). To elucidate whether 6−ME−induced cell death occurs via oxidative stress mechanisms, we employed the total ROS fluorescence probe DCFH−DA to evaluate ROS levels in both HLE and HCCLM3 cells. As illustrated in Figure 2A, treatment with 6−ME markedly elevated ROS production in HCC cells. Concurrently, the administration of antioxidants, NAC and GSH, effectively mitigated the 6−ME−induced increase in ROS levels (Figure 2A). Next, to ascertain whether the ROS generated by 6−ME treatment adversely affects cell viability, we performed CCK−8 assay. As depicted in Figure 2B, antioxidant treatment significantly reduced the cytotoxicity associated with 6−ME exposure. Colony formation assays and cellular morphology analyses demonstrated that the inclusion of antioxidants markedly enhances the survival rates and preserved the morphological integrity of both HCC cell lines compared to treatment with 6−ME alone, as evidenced by a notable decrease in the number of floating cells (Figures 2C, D). To further elucidate the role of ROS in determining the fate of HCC cells, we performed flow cytometric analysis to evaluate cell death rate in HLE and HCCLM3 cells subjected to antioxidant treatment. The results indicate that NAC and GSH significantly inhibited 6−ME−induced cell death (Figure 2E), reinforcing the hypothesis that ROS upregulation is a critical mediator of HCC cell fate mediated by 6−ME. Collectively, these findings suggest that 6−ME induces HCC cell death through the upregulation of ROS levels, underscoring the importance of oxidative stress in the mechanism of action of 6−ME.

### 3.3 6−ME induces HCC cell death via ferroptosis

**FIGURE 3:**
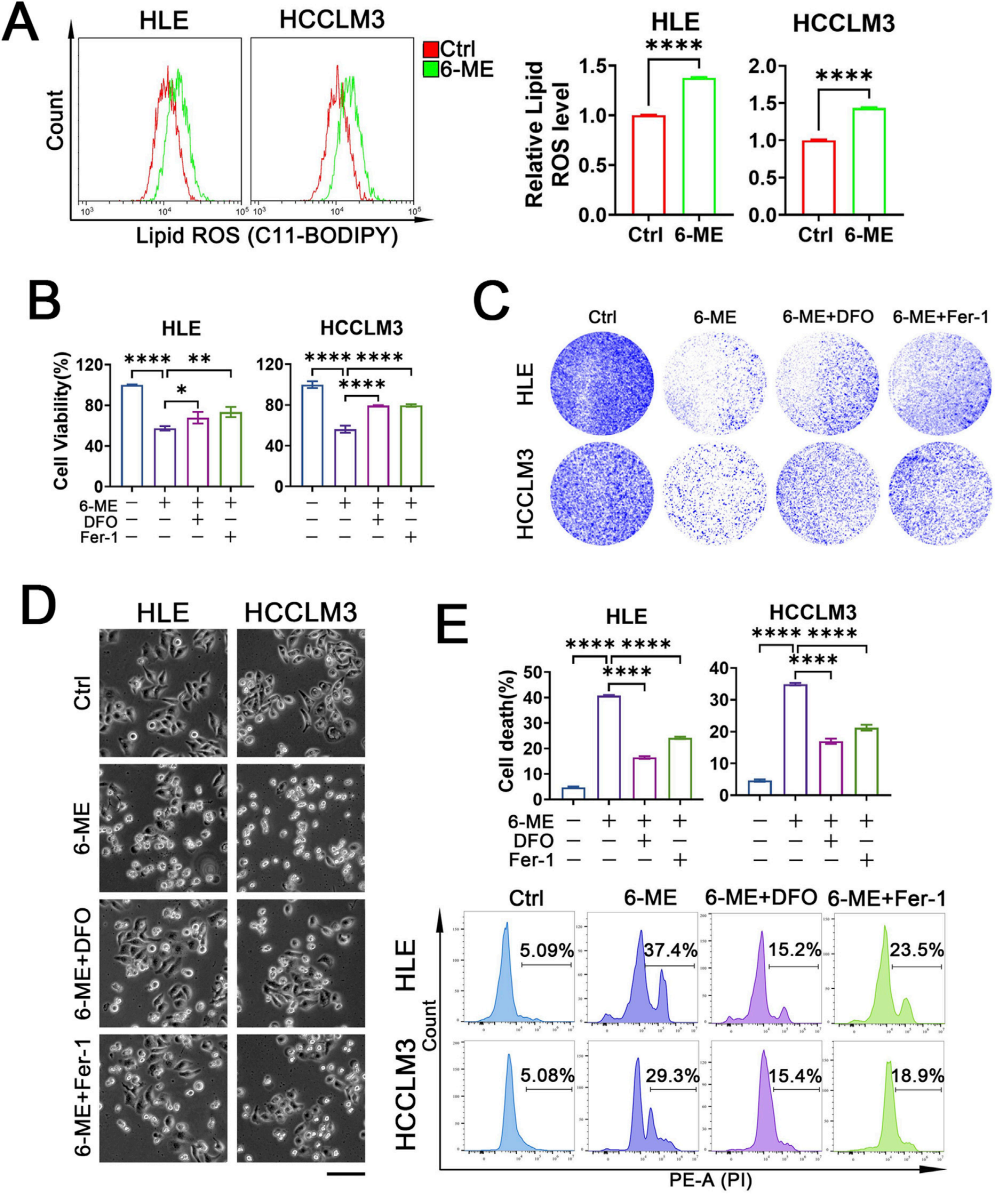
6−ME induces HCC cell death via ferroptosis. **(A−E)** HLE and HCCLM3 cells were treated with or without 6−ME (1 μM for HLE and 1.5 μM for HCCLM3) in the presence or absence of 50 μM DFO or 2 μM Fer−1 for 4 h **(A)** or 12 h **(B−E)**. **(A)** Cells were incubated with C11−BODIPY 581/591 probe for 1 h, and lipid ROS levels were evaluated using flow cytometry (n = 3). Student’s *t*−test was conducted for the statistical significance. **(B)** Cell viability was detected using CCK−8 (n = 3). **(C)** The clonogenic potential was assessed through crystal violet staining. **(D)** The cellular morphology was observed under an inverted fluorescence microscope, scale bar: 100 μm. **(E)** Cells were stained with PI followed by cell death analysis with flow cytometry (n = 3). One−way ANOVA **(B, E)** was used for statistical analysis. Data are represented as mean ± SD. Statistical significance was determined using *p < 0.05, **p < 0.01, and ****p < 0.0001.

Our above investigation has revealed that 6−ME induces HCC cell death by up−regulating ROS levels. In recent years, emerging evidence highlights the pivotal role of ferroptosis in tumor cell death. Lipid peroxidation, a hallmark of ferroptosis, particularly the excessive oxidation of phospholipids on the cell membrane, disrupts membrane integrity, culminating in membrane rupture and subsequent initiation of ferroptosis (Koren and Fuchs, 2021). Therefore, we speculated the involvement of ferroptosis in 6−ME−induced cell demise. To test this, we assessed lipid peroxide content in HCC cells. Flow cytometry analysis revealed a significant elevation in lipid ROS levels upon 6−ME treatment, signifying its capacity to induce lipid peroxidation (Figure 3A). Subsequently, we treated HCC cells with the iron chelator DFO and the ferroptosis inhibitor Fer−1 respectively and observed the rescuing effects of each inhibitor on 6−ME−mediated decrease in cell viability. As shown in Figure 3B, both inhibitors mitigated the decline in HCC cell viability induced by 6−ME. Furthermore, colony formation assay demonstrated a notable increase in the survival rates of two distinct HCC cell lines upon ferroptosis inhibitor supplementation (Figure 3C). Consistent results were observed through morphological assessments, revealing that ferroptosis inhibitors ameliorated the morphological alterations and cell death induced by 6−ME (Figure 3D). To further confirm 6−ME−triggered ferroptosis in HCC cells, we evaluated cell death via flow cytometry. Figure 3E illustrates that the addition of DFO or Fer−1 both rescued 6−ME−induced HCC cell death. Collectively, these findings suggest that 6−ME drives HCC cell death through the induction of ferroptosis.

### 3.4 Low dose 6−ME sensitizes HCC to ferroptosis

**FIGURE 4:**
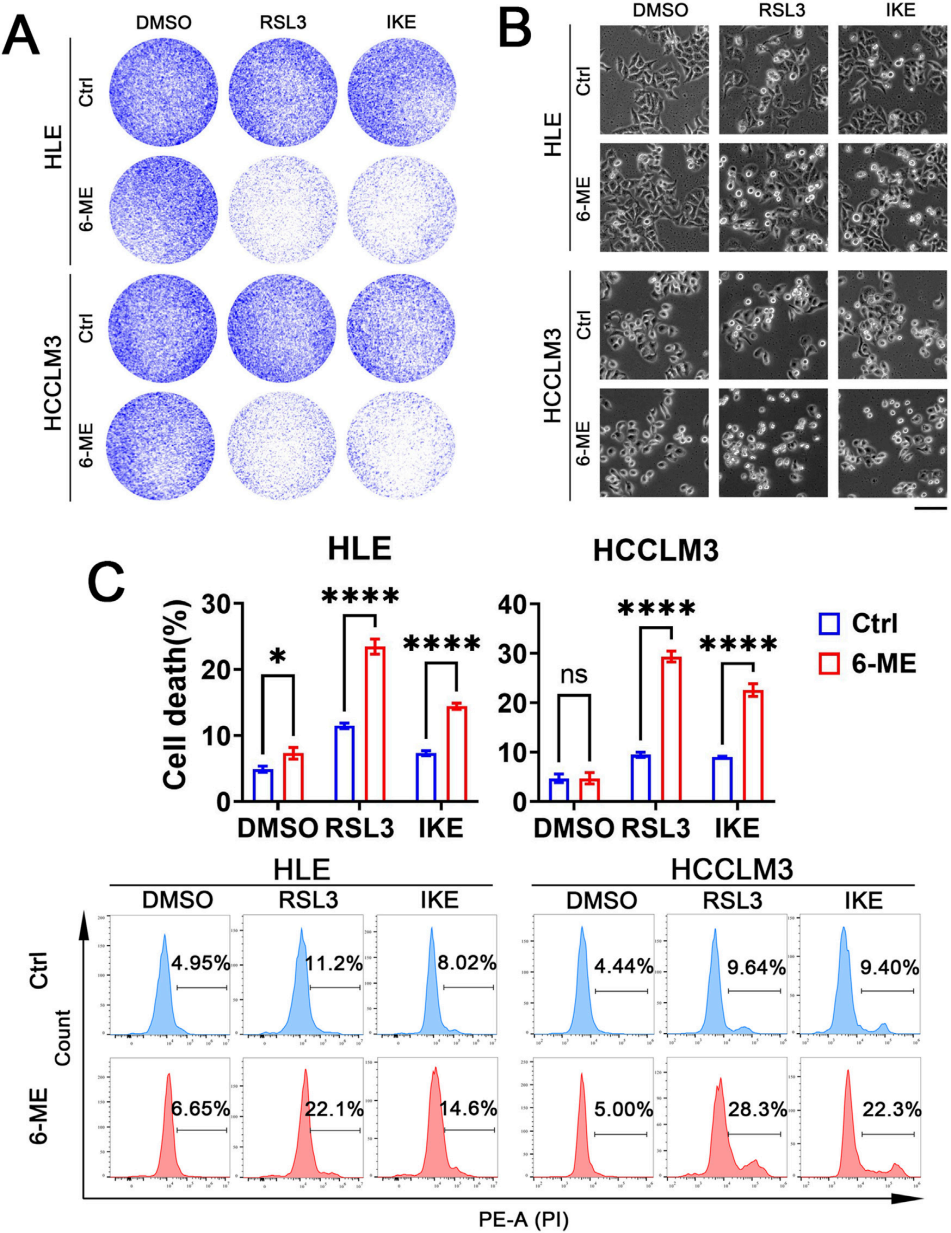
Low dose 6−ME sensitizes HCC ferroptosis. In the context of the experimental manipulation of HLE and HCCLM3 cells with or without 6−ME (0.5 μM for HLE and 0.75 μM for HCCLM3) pretreatment, cells were concurrently exposed to a combination of 2.5 μM RSL3 or 10 μM IKE for 16 h. **(A)** The clonogenic potential was assessed utilizing crystal violet staining methodology. **(B)** The cellular morphology was examined, scale bar: 100 μm. **(C)** Cell death was evaluated after PI staining via flow cytometry, n = 3. One−way ANOVA was used for statistical analysis. Data are represented as mean ± SD. Statistical significance is indicated by asterisks as follows: ns, no significance, *p < 0.05, and ****p < 0.0001.

The development of chemotherapy resistance in HCC can result in liver injury due to severe adverse reactions, hampering the efficacy of chemotherapy for HCC. Thus, there is an urgent need to identify targets that can potentiate the sensitivity of HCC cells to chemotherapeutic agents and devise collaborative strategies to mitigate the side effects and drug resistance associated with chemotherapy (Zhan et al., 2023). Our above study has demonstrated that low concentrations of 6−ME alone are insufficient to induce significant cell death in HLE and HCCLM3 cells. Consequently, we further investigated whether low doses of 6−ME could potentiate ferroptosis in HCC cells triggered by ferroptosis canonical inducers such as RSL3 or IKE. HLE and HCCLM3 cells were treated with low concentrations of 6−ME in combination with or without RSL3 or IKE, respectively. Colony formation assay and cellular morphology analysis confirmed enhanced morphological alterations and increased cell death in HLE and HCCLM3 cells following the combined low 6−ME concentrations with RSL3 or IKE treatment (Figures 4A, B). Importantly, flow cytometry analysis also demonstrated a substantial augmentation of cell death in HLE and HCCLM3 cells upon exposure to low 6−ME concentrations in conjunction with RSL3 or IKE (Figure 4C). These findings suggest that the synergistic action of low 6−ME concentrations with RSL3 or IKE can augment the susceptibility of HCC cells to ferroptosis, highlighting a potential strategy to enhance chemosensitivity in HCC therapy.

### 3.5 6−ME inhibits GPX4 transcription

**FIGURE 5:**
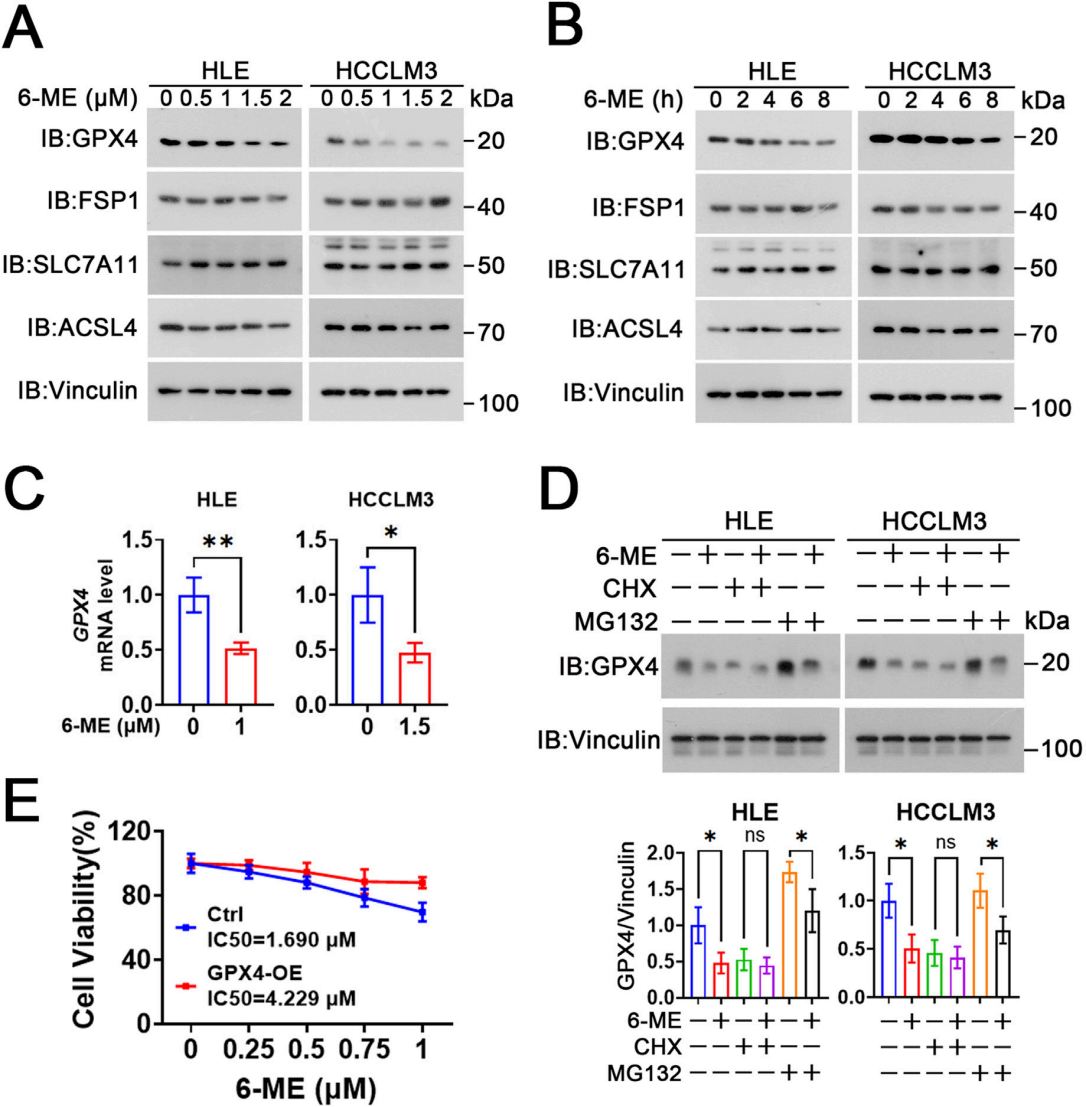
6−ME inhibits GPX4 transcription. **(A)** After different concentrations of 6−ME (0, 0.5, 1, 1.5, 2 μM) treatment for 9 h, the expression of ferroptosis−associated proteins in HLE cells and HCCLM3 cells were detected by Western blot with indicated antibodies, Vinculin as a loading control. **(B)** After HLE and HCCLM3 cells were treated with different concentrations of 6−ME (1 μM for HLE and 1.5 μM for HCCLM3) for the indicated time, the expressions of ferroptosis−associated proteins were detected by Western blot. **(C)** HLE and HCCLM3 cells were treated with the indicated concentrations of 6−ME for 6 h. The mRNA expression of *GPX4* was detected by RT−qPCR (n = 3). **(D)** HLE and HCCLM3 cells were treated with different concentrations of 6−ME (1 μM for HLE and 1.5 μM for HCCLM3) for 2 h, followed by the addition of 100 μg/mL CHX or 10 μM MG132 for 6 h. The relative protein expression of GPX4 was measured by Western blot, Vinculin as a loading control (n = 3). **(E)** HLE cells were transiently transfected with or without GPX4, followed by treatment with 6-ME for 12 h. Cell viability was quantified using the CCK-8 assay (n = 3). Data are represented as mean ± SD. Student’s *t*−test **(C, D)** was used for statistical analysis. *p < 0.05, **p < 0.01, and ns: no significance.

To elucidate the effect of 6−ME on ferroptosis in HCC cells, we evaluated the expression of ferroptosis−related proteins (ACSL4, GPX4, SLC7A11 and FSP1) in HCC cells after 6−ME treatment. Western blot analyses revealed that the expression of ACSL4, SLC7A11 and FSP1 did not exhibit significant alterations in HLE and HCCLM3 cells when compared to the control cells across different concentrations (0.5, 1, 1.5, 2 μM) and time points (2, 4, 6, 8 h) of 6−ME exposure. Conversely, the protein levels of GPX4 were downregulated in a dose− and time−dependent manner following 6−ME administration (Figure 5A, B). Additionally, we further examined the mRNA levels of GPX4 in HLE and HCCLM3 cells following treatment with 6−ME. RT−qPCR analyses reveal that 6−ME treatment significantly decreases the GPX4 mRNA level, implying that 6−ME may downregulate GPX4 expression at transcriptional level (Figure 5C). CHX is the most widely used inhibitor of protein synthesis that blocks translation by interfering with the translocation step in protein elongation (Schneider-Poetsch et al., 2010). MG132 is a potent and reversible proteasome inhibitor that prevents the degradation of ubiquitinated proteins in the proteasome (Harhouri et al., 2017). In our study, we found that the presence of CHX did not alter GPX4 expression after 6−ME treatment in HCC cells. In contrast, when MG132 was administered, 6−ME still caused downregulation of GPX4 protein levels in HCC cells. These findings suggest that the negative regulation of GPX4 by 6−ME occurs at the transcriptional level rather than through the proteasome pathway in HCC cells (Figure 5D). Furthermore, overexpression of GPX4 significantly attenuated 6-ME-induced suppression of cell viability in HLE cells. (Figure 5E). Taken together, these findings suggest that 6−ME inhibits GPX4 transcription, eventually induces ferroptosis in HCC cells.

## 4 Discussion

In this study, we elucidated that the monomeric compound 6−ME elicits ferroptosis through the suppression of GPX4 expression, resulting in a marked decrement in the viability capacity of HCC cell lines. Moreover, we observed that low concentrations of 6−ME potently augmented the susceptibility to ferroptosis induced by RSL3 and IKE, thereby holding potential promise for mitigating chemotherapeutic drug resistance. This phenomenon underscores the therapeutic potential of 6−ME as a novel modulator of ferroptotic pathways in the context of HCC management.

HCC is one of the most common life−threatening liver cancers (Bian et al., 2017; Hou et al., 2020). The pathophysiology of HCC is characterized by a high degree of malignancy, insidious onset, a propensity for rapid progression, delayed diagnosis, and premature recurrence in the clinical milieu. A significant proportion of patients are diagnosed at an advanced stage, which presents a substantial challenge for therapeutic intervention (Wang and Wei, 2020). Surgical resection and orthotopic liver transplantation remain the primary non−pharmacological strategies, but these procedures are beset by issues such as a high incidence of recurrence, technical complexity, and organ scarcity. However, pharmacological treatments such as sorafenib, are plagued by drug resistance and have suboptimal prognostic outcomes (Zhou et al., 2024). Therefore, it is imperative to identify a pharmacological agent with minimal adverse effects and marked therapeutic efficacy to treat HCC and enhance the life quality of affected individuals. Accumulating evidence suggests that certain extracts derived from traditional Chinese herbal medicines exhibit potent anti−tumor properties with reduced cytotoxicity (Liao et al., 2022). 6−ME, a natural alkaloid isolated from *Macleaya cordata*, has demonstrated cytotoxicity against a variety of tumor cell lines, including HT29, HepG2, MCF−7 and SF−268 cells (Yin et al., 2005; Wang et al., 2023; Zhang L. et al., 2023). However, its efficacy against additional tumor cell types remains to be fully elucidated. In this study, we found that 6−ME can effectively inhibit the proliferation of HLE and HCCLM3 cells and 6−ME might be a potential therapeutic agent for HCC patients. Of note, 6−ME−induced cell death in HCC cells dependents on ROS generation. These observations underscore the pivotal role of ROS in the cytotoxic effects of 6−ME on HCC cells.

Ferroptosis is a new type of cell death reported for the first time in 2012, mainly caused by lipid peroxidation (Stockwell, 2022). Long−chain acyl−CoA synthetase 4 (ACSL4) and lysophosphatidylcholine acyltransferase 3 (LPCAT3) are pivotal mediators in this process, facilitating the incorporation of PUFAs into phospholipids, thereby generating PUFAs in phospholipid form (PUFA−PLs) (Doll et al., 2017). These PUFA−PLs are particularly vulnerable to free radical−induced oxidation mediated by lipoxygenases (ALOXs), resulting in iron−dependent formation of reactive oxygen species (ROS), ultimately leading to disruption of lipid bilayers and compromised membrane integrity, thereby promoting ferroptosis (Doll et al., 2017; Zou et al., 2019). In addition, this cytotoxic process is typically initiated by the inhibition of solute carrier family 7 member 11 (SLC7A11), a crucial cystine−glutamate antiporter, and glutathione peroxidase 4 (GPX4), a pivotal antioxidant enzyme. xCT/SLC7A11 is a specific light chain subunit responsible for the transport of extracellular cystine into the cell for the production of GSH, which can inhibit ferroptosis and protect cells from oxidative stress (Koppula et al., 2018). GPX4, a key inhibitor of lipid peroxidation, oxidizes glutathione (GSH) to glutathione disulfide (GSSG), which inhibits GPX4 and reduces the ability to neutralize lipid peroxidation through GSH (Maiorino et al., 2018). Also, FSP1 functions by reducing ubiquinone to ubiquinol or vitamin K to hydroquinone (VKH2), thereby inhibiting ferroptosis (Lv et al., 2023). Interestingly, our study found that 6−ME significantly increased the lipid ROS level of HLE and HCCLM3 cells. Furthermore, the ferroptosis inhibitor Fer−1 and iron chelator DFO were found to rescue the viability and ameliorate the death phenotype of 6−ME−exposed HLE and HCCLM3 cells. Most importantly, we examined the effect of 6−ME on the expression of ferroptosis−related proteins and found that 6−ME downregulated the protein level of GPX4 in a dose− and time−dependent manner.

The turnover rate of GPX4 is crucial for its role in cellular antioxidant defense and the regulation of lipid peroxidation, particularly in the context of ferroptosis. Sun et al. demonstrated that under oxidative stress, GPX4 is regulated through ubiquitination and proteasomal degradation, impacting cellular resistance to ferroptosis (Sun et al., 2023). Additionally, the Nrf2 signaling pathway modulates GPX4 expression and turnover, enhancing its stability under oxidative conditions (Xu et al., 2024). GPX4’s high turnover number allows it to efficiently detoxify lipid peroxides, further underscoring its importance in cellular protection against oxidative damage and ferroptosis (Zhang X. D. et al., 2023). These compelling results indicate that ferroptosis plays a pivotal role in the 6−ME−induced cell death of HCC cells through the suppression of GPX4 expression.

Natural products are a primary source for the development of new drugs, and compared to synthetic compounds, they typically exhibit lower toxicity and fewer side effects (Nisar et al., 2022). Prolonged administration of chemotherapeutic agents is frequently associated with the development of drug resistance, a phenomenon that compromises the efficacy of cancer treatment regimens. For example, sorafenib is a molecularly targeted drug that can be used for advanced HCC treatment, but its limited survival benefit suggests the presence of primary and acquired sorafenib resistance mechanisms in HCC cells (Sun et al., 2016). To mitigate this impediment, the strategic employment of drug combinations has emerged as a conventional approach to curtail the resistance mechanisms of chemotherapeutic agents. In our study, we subjected HLE and HCCLM3 cells to low concentrations of 6−ME, in conjunction with similarly low doses of RSL3 or IKE. Our findings demonstrated that the combinatory regimen of 6−ME with RSL3 or IKE at these reduced concentrations exerted a pronounced synergistic effect, significantly augmenting the susceptibility of HCC cells to ferroptosis. These observations suggest a promising therapeutic strategy for patients with HCC *via* the combination of 6−ME with other ferroptosis inducers.

In contrast to canonical ferroptosis inducers such as erastin and RSL3, which operate through singular molecular targets, 6−ME exhibits a multi−modal mechanism of action, concurrently depleting glutathione (via ROS−dependent oxidative stress amplification) and suppressing the PI3K/AKT/mTOR pro−survival signaling axis (Zhang L. et al., 2023). This polypharmacological activity likely underpins its enhanced cytotoxic potency (IC50 < 2 μM in HCC models) relative to natural compounds such as curcumin (IC50 ∼20 μM) or artemisinin derivatives, which require metabolic activation or exhibit limited target specificity (Chen et al., 2020; Li et al., 2022). Notably, 6−ME demonstrates synergistic interactions with both RSL3 and IKE, suggesting its capacity to bypass resistance mechanisms inherent to monotherapeutic ferroptosis induction. Despite its robust *in vitro* efficacy against HCC cells, the clinical translation of 6−ME necessitates resolution of two critical challenges. The first challenge is bioavailability limitations. Like many natural compounds, 6−ME suffers from suboptimal solubility and rapid hepatic metabolism, which may compromise its therapeutic index. Structural modifications (e.g., hydroxylation or glycosylation) or encapsulation within nanoparticle−based delivery systems (e.g., lipid−polymer hybrids) could improve its pharmacokinetic stability and tumor−targeted distribution. The second obstacle is off−target toxicity risks, while 6−ME selectively induces ferroptosis in HCC cells *in vitro*, its iron−chelation properties and ROS−generating capacity raise concerns about toxicity in iron−rich normal tissues (e.g., hepatocytes, splenic macrophages). Comprehensive *in vivo* safety assessments, including histopathological evaluation of liver/spleen and serum ferritin monitoring, are imperative to delineate its therapeutic window.

## 5 Conclusion

**FIGURE 6:**
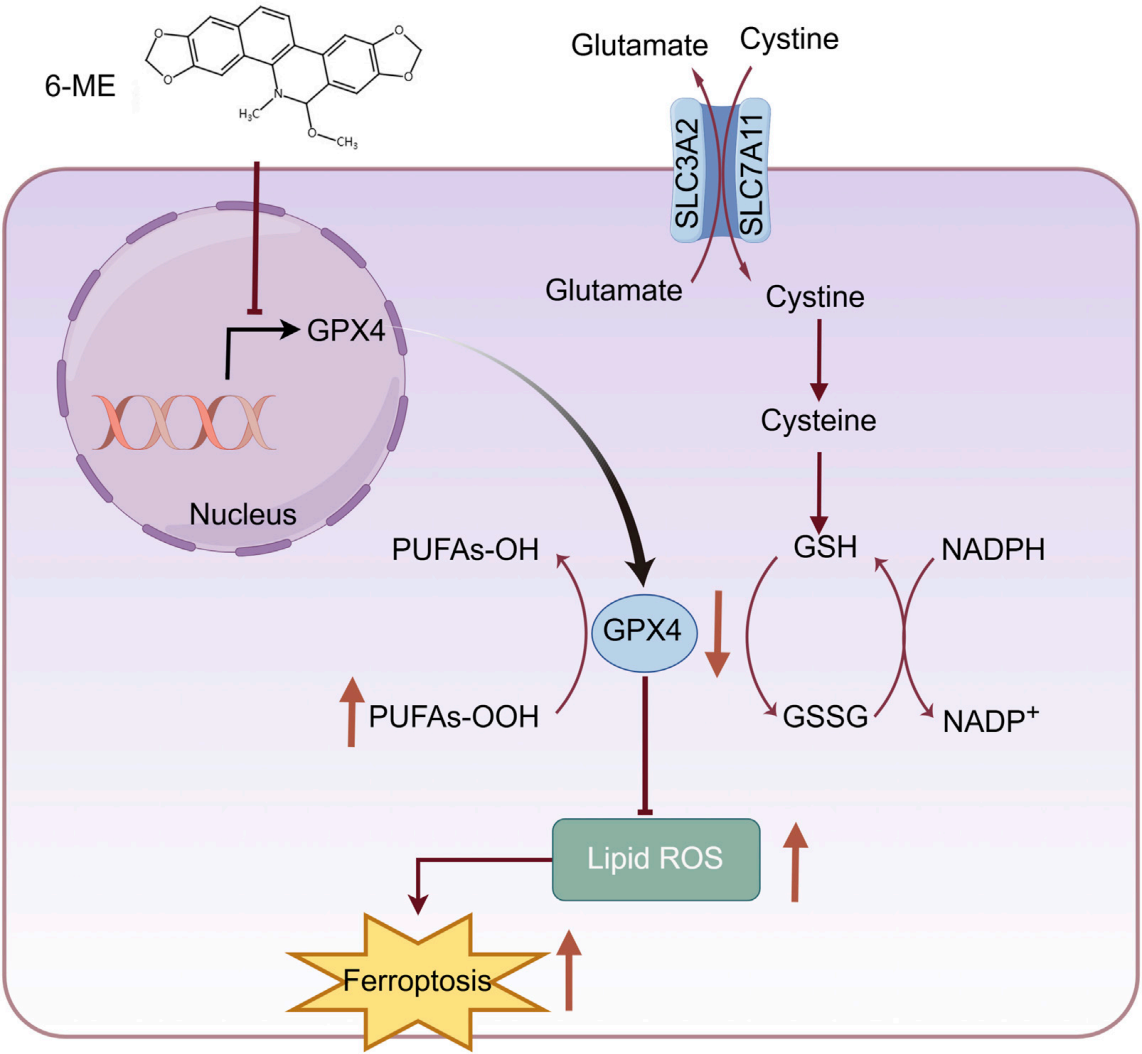
Schematic diagram showing that 6−ME induces ferroptosis in hepatocellular carcinoma cells through reducing GPX4. 6−ME increases lipid ROS through the reduction of GPX4, which leads to the ferroptosis of HCC cells. The figure was created by Figdraw (www.figdraw.com).

In summary, 6−ME inhibits GPX4 protein expression at transcriptional level, leading to an indirect elevation of lipid peroxidation, ultimately promoting ferroptosis in HCC cells (Figure 6). Moreover, low concentrations of 6−ME sensitizes HCC cells to RSL3 and IKE−induced ferroptosis, demonstrating its beneficial anti−tumor effects. This underscores 6−ME as a promising therapeutic agent for HCC. Taken together, our results suggest that 6−ME exerts its anti−HCC effects through GPX4 and the precise regulatory mechanisms require further investigation.

## Data Availability

The original contributions presented in the study are included in the article/supplementary material, further inquiries can be directed to the corresponding author.
